# Treatment intensity, age and outcome in medical ICU patients: results of a French administrative database

**DOI:** 10.1186/s13613-016-0107-y

**Published:** 2016-01-14

**Authors:** Vincent Peigne, Dominique Somme, Emmanuel Guérot, Emilie Lenain, Gilles Chatellier, Jean-Yves Fagon, Olivier Saint-Jean

**Affiliations:** Medical ICU, Hôpital Européen Georges Pompidou, Paris, France; Université Paris Descartes, Paris, France; Geriatrics Department, CHU de Rennes, Hôpital Pontchaillou, 2 rue Henri Le Guilloux, 35033 Rennes Cedex 9, France; Université de Rennes 1, Rennes, France; Clinical Research Unit, Hôpital Européen Georges Pompidou, Paris, France; Geriatrics Department, Hôpital Européen Georges Pompidou, Paris, France

**Keywords:** Intensive care, Older patients, Mechanical ventilation, Shock, Renal failure, Treatment intensity

## Abstract

**Background:**

Intensive care unit (ICU) patients are aging, and older age has been associated with higher mortality in ICU. As previous studies have reported that older age was also associated with less intensive treatment, we investigated the relationship between age, treatment intensity and mortality in medical ICU patients.

**Methods:**

Data were extracted from the administrative database of 18 medical ICUs. Patients with a unique medical ICU stay and a Simplified Acute Physiology Score II (without age-related points) >15 were included. Treatment intensity was described with a novel indicator, which is a four-group classification based upon the most frequent ICU procedures. The relationship between age, treatment intensity and hospital mortality was analyzed with the estimation of standardized mortality ratio in the four groups of treatment intensity.

**Results:**

A total of 23,578 patients, including 3203 patients aged ≥80 years, were analyzed. Hospital mortality increased from 13 % for the younger patients (age < 40 years) to 38 % for the older patients (age ≥ 80 years), while Simplified Acute Physiology Score II (without age-related points) increased only from 36 (age < 40 years) to 43 (age ≥ 80). Hospital mortality increased with age in the four groups of treatment intensity. Standardized mortality ratio increased with age among the patients with less intensive treatment but was not associated with age among the patients with the highest treatment intensity.

**Conclusion:**

Our results support the fact that the increase in mortality with age among ICU patients is not related to an increase in severity. Using a new tool to estimate ICU treatment intensity, our study suggests that mortality of ICU patients increases with age whatever the treatment intensity is. Further investigations are required to determinate whether this increase in mortality among older ICU patients is related to undertreatment or to a lower efficiency of organ support treatment.

## Background

Intensive care unit (ICU) patients are aging as the global population does, with, as an example, 13 % of patients >80 years among 120,123 ICU patients in a recent study from Australia and New Zealand [1]. Management of critically ill old patients will be a more and more frequent task for physicians in the next decades.

The relationship between age and prognosis of ICU patients has been extensively analyzed among the last 30 years. Older age was associated with a higher mortality in large prospective studies [2–5]. Similar findings were made in smaller studies investigating the outcome of subgroups of patients with various organ failures [6–8].

However, many uncertainties remain because these issues are complex and evolving. Indeed, the definition of an “elderly patient” is equivocal. The age thresholds used in the literature to consider a patient as an elderly one vary from 60 to 80 years [9, 10]. Another pitfall in the analysis of the published studies is the increase in the life expectancy in the global population during the last decades. The elderly patients admitted in ICU 20 years ago had a lower life expectancy than those admitted now. The current validity of previously published studies is questionable. Another, and probably major, bias in the analysis of the impact of age on outcome is the variability of the behaviors of the physicians in charge of elderly patients. Elderly patients are less admitted in ICU than younger critically ill patients [11–13] and may receive less intensive treatment [14], and their treatments are more frequently withheld [15]. Medical management of elderly critically ill patients evolved during the latest decade with increased ICU admission and increased ICU caregivers’ workload [16]. Data about treatment intensity and age are scarce and ambiguous because well-established measurements of treatment intensity are still lacking. Therapeutic Intervention Scoring System (TISS-28) [17], OMEGA [18] and Nine Equivalents of nursing Manpower use Score (NEMS) [19], the most commonly used scores, are derived from the TISS score, which was developed as a severity index. These scores are validated to measure the ICU staff workload and not to evaluate the therapeutic intensity. The European Society of Intensive Care Medicine (ESICM) defined three levels of care (LOC) to describe the ICUs [20]. These ESICM’s LOCs take into account the number of organ failure that can be supported in the unit. Such an approach is used by the French National Health Insurance System to define the level of payment of ICU stays.

For these reasons, we designed the present study to describe the relationship between age and outcome of current ICU patients according to the therapeutic intensity.

## Methods

### Study population

We analyzed the administrative database of the Assistance Publique—Hôpitaux de Paris. Our institution is made of 37 public teaching hospitals and includes 18 medical ICUs. Medical and administrative data from each hospital are merged in a central database. Since 2004, French intensivists are required to code for significant procedures (as defined by the French National Health Insurance System) and for Simplified Acute Physiology Score (SAPS2) [2]. Noncoding of these data has a negative financial impact upon hospital’s financing. Quality controls are locally performed to detect over-coding and under-coding.

The data collected for each hospital stay include age, gender, type and number of the performed procedures, the identification and the length of stays in the different units of the hospital. Diagnoses and comorbidities were coded according to the International Classification of the Diseases ICD-10. The diagnosis that required the higher resource consumption during the ICU stay was quoted, but the admission diagnosis was not specifically identified. The coding process is regularly updated.

Data are anonymous, and patients with multiple hospital stays cannot be identified.

Hospital stays with one medical ICU stay and an age-adjusted SAPS2 > 15 (threshold used in France to separate intensive care and intermediate care patients) were included in the analysis. Cases with multiple ICU stays during the same hospital stay or with unknown SAPS2 cases were excluded. Period of analysis was 2006–2008 because the coding process was similar during these 3 years.

### Treatment intensity classification

A treatment intensity indicator was built by an expert consensus based on the French National Health Insurance System criteria of level of payment of ICU stays (Table 1). The indicator took into account the most frequent procedures: hemodynamic support, respiratory support and renal support. Two levels of support were defined: low-intense (hemodynamic: use of less than 8 μg/kg/min of dobutamine or dopamine; respiratory: noninvasive ventilation or mechanical ventilation with FiO2 < 0.6 and PEEP < 6 cm H2O) and high-intense (hemodynamic: use of more than 8 μg/kg/min of dobutamine or dopamine, or use of epinephrine or norepinephrine; respiratory: mechanical ventilation with FiO2 ≥ 0.6 or PEEP ≥ 6 cm H2O; renal: any type of renal replacement therapy). A four-group treatment intensity classification was defined according to the number and the level of supports: no support (group 0), only one low-intense support (group 1), two low-intense supports or one high-intense support (group 2) and more intense support (group 3).

**Table 1:** Treatment intensity indicator

Supports	Groups of treatment intensity
Low-intense	
Dobutamine or dopamine ≤8 µg/kg/min	0: no support
Noninvasive ventilation	1: one low-intense support
Mechanical ventilation with FiO2 < 0.6 and PEEP < 6 cm H2O	2: two low-intense supports or one high-intense support
	3: more intense support
High-intense	
Dobutamine/dopamine >8 µg/kg/min, epinephrine or norepinephrine	
Mechanical ventilation with FiO2 ≥ 0.6 or PEEP ≥ 6 cm H2O	
Renal replacement therapy	

### Statistical analysis

SAPS2 is a prospectively validated severity score [2], based upon physiologic data. The score can be converted to a probability of hospital mortality [2]. This probability of hospital mortality has been widely used to determinate standardized mortality ratio (SMR) between observed mortality and SAPS2-expected mortality in order to analyze the performance of ICUs. Fifteen variables, including age, are required to calculate SAPS2. Age-adjusted SAPS2 is calculated by subtracting the age-related points and is commonly used in the literature [21]. Age-adjusted SAPS2 was used as an age-independent severity index. Standardized mortality ratio (SMR) was calculated using SAPS2. Age was analyzed as a continuous, a qualitative (the age categories of the SAPS2 score) and a binary (<80 or ≥80 years) variable. Quantitative data were presented as means and standard deviation (SD). Their variations were analyzed using the ANOVA. Qualitative data are presented as numbers and percentages. Their variations were analyzed using the Chi-square test. Statistical analysis was performed with the STATA software (StataCorp, Texas).

## Results

### Study population

**Fig. 1:**
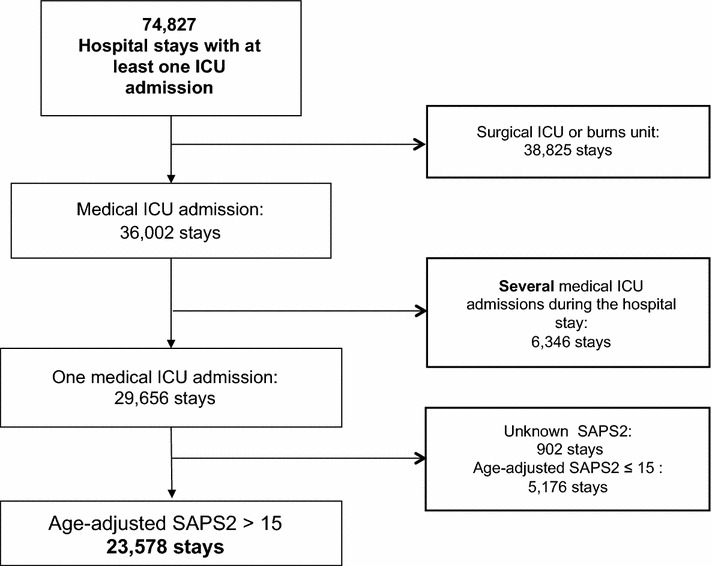
Flowchart

During the 3-year period of interest, 23,578 stays met the inclusion criteria and were analyzed (Fig. 1: flowchart).

Sex ratio was 1.47 (14,023 men/9555 women). Mean age was 58.3 years (SD 18.2), and 3203 (13.6 %) patients were ≥80 year old. Mean age-adjusted SAPS2 was 41 (SD 21). Hospital mortality rate was 29.5 %.

### Severity and mortality according to the age

**Fig. 2:**
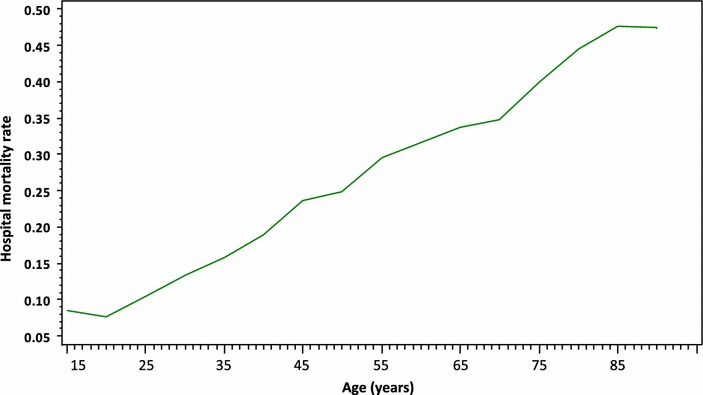
Hospital mortality and age

Mean age-adjusted SAPS2 increased slightly but significantly with age: 36 (SD 18) in <40-year-old patients, 41 (SD 20) in 40- to 69-year-old patients, 41 (SD 21) in 70- to 74-year-old patients, 42 (SD 22) in 75- to 79-year-old patients and 43 (SD 22) in patients older than 80 years (*P* < .001). Crude hospital mortality increased with age, as illustrated in Fig. 2, from 13 % (SD 33) in <40-year-old patients, 23 % (SD 42) in 40- to 69-year-old patients, 32 % (SD 47) in 75- to 79-year-old patients and 38 (SD 49) in patients older than 80 years (*P* < .001).

### Treatment intensity and age

Among the 23,578 patients analyzed, 7297 (31 %) did not receive any hemodynamic, ventilator or renal support (group 0), 2961 (14.6 %) received only one low-intense support (group 1), 3212 (13.6 %) received two low-intense supports or one high-intense support (group 2) and 10,108 (42.9 %) received more intense support (group 3).

Mean age differed slightly but significantly (*P* < .001) across the four groups: 58.6 (SD 19.3) years for group 0, 54.0 (SD 18.7) for group 1, 59.5 (SD 18.2) for group 2 and 61.3 (SD 16.8) for group 3. The distribution of treatment intensity was different (*P* < 0.001) in the SAPS2 age subgroups, as reported in Table 2. The distribution of treatment intensity in the patients aged <80 and ≥80 years is reported in Table 3 (*P* < 0.001).

**Table 2:** Treatment intensity in the different SAPS2 age subgroups

	<40 years [*n* (%)]	40–59 years [*n* (%)]	60–69 years [*n* (%)]	70–74 years [*n* (%)]	75–79 years [*n* (%)]	≥80 years [*n* (%)]
Group 0	1381 (35.6 %)	2298 (29.7 %)	1170 (28.5 %)	643 (28.8 %)	732 (30.1 %)	1073 (33.5 %)
Group 1	776 (20.0 %)	1012 (13.1 %)	445 (10.8 %)	232 (10.4 %)	230 (9.5 %)	266 (8.3 %)
Group 2	520 (13.4 %)	1066 (13.8 %)	546 (13.3 %)	315 (14.1 %)	308 (12.7 %)	457 (14.3 %)
Group 3	1200 (31.0 %)	3351 (43.4 %)	1951 (47.4 %)	1040 (46.6 %)	1159 (47.7 %)	1407 (43.9 %)

Comparison of the distributions by the Kruskal–Wallis test *P* < .001

**Table 3:** Treatment intensity in the patients aged <80 and ≥80 years

	<80 years [*n* (%)]	≥80 years [*n* (%)]
Group 0	6624 (30.5 %)	1073 (33.5 %)
Group 1	2695 (13.2 %)	266 (8.3 %)
Group 2	2755 (13.5 %)	457 (14.3 %)
Group 3	8701 (42.7 %)	1407 (43.9 %)

### Treatment intensity, age and mortality

Hospital mortality significantly increased with treatment intensity: 10.1 % in group 0, 15.6 % in group 1, 19.3 % in group 2 and 50.7 % in group 3 (*P* < .001).

**Fig. 3:**
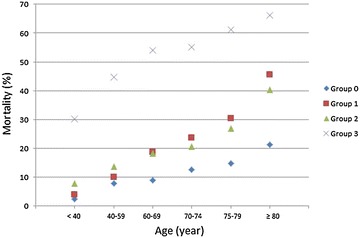
Hospital mortality according to treatment intensity and age

Hospital mortality significantly increased with age in all treatment intensity groups (Table 4; Fig. 3). Patients aged ≥80 years had higher mortality than the patients aged <80 in the four treatment intensity groups: group 0 21.3 versus 8.2 % (*P* < .001), group 1 45.5 versus 12.6 % (*P* < .001), group 2 40.3 versus 15.8 % (*P* < .001) and group 3 66.2 % versus 48.2 % (*P* < .001).

**Table 4:** Hospital mortality according to the age of the patients in the four groups of treatment intensity

	All ages [*n* (%)]	<40 years [*n* (%)]	40–59 years [*n* (%)]	60–69 years [*n* (%)]	70–74 years [*n* (%)]	75–79 years [*n* (%)]	≥80 years [*n* (%)]	*P*
Group 0	738 (10.1 %)	35 (2.5 %)	180 (7.8 %)	105 (9.0 %)	81 (12.6 %)	108 (14.8 %)	229 (21.3 %)	<.001
Group 1	461 (15.6 %)	30 (3.9 %)	102 (10.1 %)	83 (18.7 %)	55 (23.7 %)	70 (30.4 %)	121 (45.5 %)	<.001
Group 2	619 (19.3 %)	41 (7.9 %)	146 (13.7 %)	100 (18.3 %)	65 (20.6 %)	83 (26.9 %)	184 (40.3 %)	<.001
Group 3	5128 (50.7 %)	362 (30.2 %)	1496 (44.6 %)	1056 (54.1 %)	574 (55.2 %)	709 (61.2 %)	931 (66.2 %)	<.001

### Standardized mortality ratio (SMR) according to age and treatment intensity

**Fig. 4:**
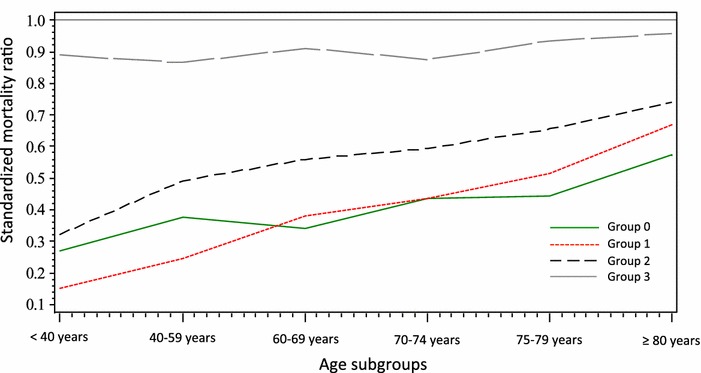
Relationship between the standardized mortality ratio (SMR = observed mortality/expected mortality) and age in the four groups of treatment intensity. SMR increased with age in the lower intensity groups, reflecting either inadequate treatment intensity or a lower efficiency of treatment for the older patients. SMR was not related to age in the highest intensity group, reflecting a similar efficiency of such treatment whatever was the age of the patients

SMR increased with age among patients of group 0, group 1 and group 2 but not in group 3 (Fig. 4). SMR of the younger patients was very low in group 0, group 1 and group 2.

## Discussion

This study describes more than 23,000 hospital stays including a unique medical ICU admission at the Assistance Publique—Hôpitaux de Paris from January 1, 2006, to December 31, 2008. Patients aged ≥80 years were involved in more than 3200 stays. Hospital mortality increased with age, as previously reported. This increase in mortality with age was not related to an increase in severity (reflected by age-adjusted SAPS2).

Another point in the study is the use of a novel tool to describe treatment intensity by integrating data from the administrative database of the largest European hospital organization. This indicator was not designed to describe severity but to estimate the amount of care delivered to the patients. We could establish that mortality increased with age whatever the treatment intensity was. Several hypotheses should be discussed to explain this fact: (1) some differences in treatment allocation with elderly patients receiving less sustained intensive treatment, (2) different ICU admission policies according to age and (3) a decreased efficiency of organ support in elderly patients.

The first hypothesis cannot be excluded because of the lack of data about treatment withdrawal in the administrative database used in our study. The high mortality (more than 20 %) among patients ≥80 years admitted in the ICU who did not receive vasopressors, mechanical ventilation or renal replacement is suggestive of “do not resuscitate” decision in some of these patients. The distribution of treatment intensity was different within the different age groups, but the differences seemed to be clinically insignificant and reflected mainly the great size of the study population. Indeed, the proportion of group 0 and group 3 were quite similar among patients aged ≥80 years and <80 years (33.5 and 30.5 % for group 0 and 43.9 versus 42.7 % for group 3). This result suggests that the increase in mortality with age was not, or not only, related to a difference of treatment intensity and that other explanations should be discussed.

Admission policies had not been investigated specifically in the present study that analyzed only hospital stays of patients admitted once in a medical ICU. Data about the patients who were denied ICU admission are lacking. No data were available about comorbidities and functional status. However, severity was not related to age, suggesting that ICU patients were quite homogenous.

The hypothesis of a decreased efficiency of organ support in elderly patients is strengthened by the increase in mortality with age in the four groups of treatment intensity and by the increase in SMR with age in the group 0, group 1 and group 2. This hypothesis cannot be confirmed because treatment intensity could be inadequate in some patients (“do not resuscitate” orders) and because the timeline of the treatment was not investigated. Indeed, ICU treatments could be less effective if they are delayed [22]. However, the absence of variation of SMR according to the age in the patients receiving the most intensive treatment (group 3) suggests that the efficiency of organ support is not decreased in selected elderly patients. Our results are homogenous with a recent study, which analyzed the amount of care required per survivor in ICU patients. Elderly patients required a higher amount of care per survivor than the younger patients, except for the most critically ill patients [23]. The very low SMR of the younger patients is amazing and may be explained by a higher adequacy of level of care and by a more liberal admission policy.

The present study faced several difficulties. The first difficulty was how to define who the elderly patients are. For this reason, age was analyzed by using three different approaches: as a continuous variable, as a categorical variable (use of the subgroups of the SAPS2 score) and as a dichotomized variable with an elevated threshold (80 years) already used in the literature [1, 14]. Similar results were obtained with these three approaches. The administrative database used in the present study did not procure enough information to describe the elderly patients with more precise tools like frailty indexes. Another difficulty was the measurement of the treatment intensity. The currently used scores, TISS-28, NEMS and OMEGA, provide validated measurements of the ICU staff workload but have not been designed to estimate the treatment intensity. We developed an original and simple measurement based on the items selected by the French National Health Insurance System to describe the intensity of care of ICU patients. We focused on the three most common organ supports provided in ICUs: vasopressors, mechanical ventilation and renal replacement. Interestingly, these variables are already used in validated organ failure scores [24, 25]. None of these organ failure scores are implemented in the administrative database used in the present study. However, these scores cannot help us to better analyze the relationship between severity and treatment intensity because they are determined by both severity and treatment intensity. In the present study, we chose to analyze the use of organ supports as a marker of treatment intensity and not as a marker of severity.

The present study has several limits. First, data have been extracted from an administrative database. Coding accuracy was not controlled specifically for the study. In particular, some patients may have been over-treated to allow upcoding (for instance, FiO2 61 %, PEEP 7 cm H2O) [26, 27]. Data about the number of occurrences or the duration of each organ support are also lacking. Another limit was the use of hospital mortality as unique outcome criterion. Pertinent outcome indicators like functional recovery, middle-term mortality and readmission could not be evaluated. Available data did not allow us to take into account comorbidities, frailty and withholding/withdrawal of life support. Finally, multiple ICU stays during the same hospital stay were excluded from the analysis because patients requiring ICU readmission have a higher severity and a higher mortality rate than the other ICU patients [28]. About 17.6 % (6346/36,002) of ICU stays were excluded for this reason. We could not identify the patients who had recurrent hospital stays with unique ICU admission during each hospital stay.

In spite of these limits, our findings are valuable for several reasons. First, a large sample (>23,000) of hospital stays of ICU has been analyzed, including more than 3200 stays involving patients aged ≥80 years. The administrative database used in the study is regularly controlled to avoid over-coding and missing data. Hospitals are financially encouraged to code for the procedures reflecting the treatment intensity. Data came from a high number of medical ICUs. Patients treated with different medical strategies had been included, offering the opportunity to analyze the relationships between age, treatment intensity and mortality. The large number of ICUs allowed us to collect data about a huge number of patients treated during a 3-year period. The shortness of the period of data collection increased the homogeneity of the sample and limited the bias related to the decalibration of SAPS2 [21].

As a conclusion, the present study describes a new tool to estimate ICU treatment intensity and supports the fact that mortality of ICU patients increases with age whatever the treatment intensity is. Further qualitative longitudinal studies paying attention to the patients’ functional capacity and to the medical practices are required to better understand the relationship between age and mortality of ICU patients.
